# Amino Acid Substitutions in Loop C of Arabidopsis PIP2 Aquaporins Alters the Permeability of CO_2_

**DOI:** 10.1111/pce.15635

**Published:** 2025-06-03

**Authors:** Shaila Shermin Tania, Shigeko Utsugi, Yoshiyuki Tsuchiya, Shizuka Sasano, Maki Katsuhara, Izumi C. Mori

**Affiliations:** ^1^ Institute of Plant Science and Resources Okayama University Kurashiki Okayama Japan

**Keywords:** *Arabidopsis thaliana*, CO_2_ transport, monomeric pore, PIP2 aquaporin, *Xenopus laevis*

## Abstract

The transport of CO_2_ across biomembranes in plant cells is essential for efficient photosynthesis. Some aquaporins capable of CO_2_ transport, referred to as ‘COOporins’, are postulated to play a crucial role in leaf CO_2_ diffusion. However, the structural basis of CO_2_ permeation through aquaporins remains largely unknown. Here, we show that amino acids in loop C are critical for the CO_2_ permeability of *Arabidopsis thaliana* PIP2 aquaporins. We found that swapping tyrosine and serine in loop C to histidine and phenylalanine, which differ between AtPIP2;1 and AtPIP2;3, altered CO_2_ permeability when examined in the *Xenopus laevis* oocyte heterologous expression system. AlphaFold2 modelling indicated that these substitution induced a conformational shift in the sidechain of arginine in the aromatic/arginine (ar/R) selectivity filter and in lysine at the extracellular mouth of the monomeric pore in PIP2 aquaporins. Our findings demonstrate that distal amino acid substitutions can trigger conformational changes of the ar/R filter in the monomeric pore, modulating CO_2_ permeability. Additionally, phylogenetic analysis suggested that aquaporins capable of dual water/CO_2_ permeability are ancestral to those that are water‐selective and CO_2_‐impermeable, and CO_2_‐selective and water impermeable.

## Summary statement

While a role of aquaporins in CO_2_ diffusion in a leaf has been acknowledged, the structural fundamentals for CO_2_ permeation is not understood. Here, we showed that tyrosine and serine residues in the extracellular loop were involved in CO_2_ permeability of Arabidopsis PIP2 aquaporin.

## 1 Introduction

Aquaporins are integral membrane proteins initially discovered as water channels in human red blood cells (Agre et al. 1993). Found across all kingdoms of life, aquaporins play essential roles in biological systems and have been extensively studied (Chaumont and Tyerman 2014; Javot 2002; Kaldenhoff and Fischer 2006; Maurel et al. 2009; Moshelion et al. 2015). In plants, they are classified into five subfamilies based on function, subcellular localization, and primary structure: plasma membrane intrinsic proteins (PIPs), tonoplast intrinsic proteins (TIPs), small basic intrinsic proteins (SIPs), nodulin‐26 like intrinsic proteins (NIPs) and X intrinsic proteins (XIPs) (Maurel et al. 2015; Y. Wang et al. 2020). Plant aquaporins facilitate the transport of diverse substrates in addition to water, including hydrogen peroxide, boric acid, silicic acid, arsenous acid, ammonia, oxygen and monovalent cations (Byrt et al. 2017; Holm et al. 2005; Kourghi et al. 2017; Maurel et al. 2002; Zwiazek et al. 2017). Notably, some PIP aquaporins are reported to transport CO_2_ (Ermakova et al. 2021; Heckwolf et al. 2011; Mori et al. 2014; Otto et al. 2010; Uehlein et al. 2003; Uehlein, Otto, et al. 2012) and have been termed ‘COOporins’ (Mizokami et al. 2022; Terashima et al. 2006).

Atmospheric CO_2_ is the primary source for photosynthesis in terrestrial plants. It enters the leaf interior through stomatal openings and diffuses to the chloroplast stroma, where carboxylation occurs. During this process, CO_2_ traverses the stomatal pore, substomatal cavity, mesophyll cell wall, plasma membrane, cytosol, chloroplast envelope membranes and chloroplast stroma. The CO_2_ permeability of the plasma membrane of mesophyll cells is reported to be significantly lower than that of a pure lipid bilayer (Heckwolf et al. 2011). Consequently, COOporins are proposed to play a crucial role in maintaining sufficiently high CO_2_ permeability for photosynthesis. Several studies have provided evidence for their significance in mesophyll conductance (*g*_m_), based on the effect of the aquaporin inhibitor HgCl_2_ (Terashima and Ono 2002) and the phenotypes of PIP over‐expressing transgenic plants (Ermakova et al. 2021; Hanba et al. 2004; Kawase et al. 2013; Xu et al. 2019). Mesophyll conductance is the diffusion constant of CO_2_ within the leaf from the substomatal cavity to chloroplasts in mesophyll cells and reciprocal of mesophyll resistance (Flexas et al. 2008).

Despite the physiological significance suggested by studies on PIP‐overexpressing transgenic plants, the phenotypes of knock‐out mutants remain obscure. *atpip1;2* loss‐of‐function mutants exhibited reduced *g*_m_ (Uehlein, Sperling, et al. 2012), and *ospip1;2*‐knockout mutants in rice showed a role of OsPIP1;2 in *g*_m_ and growth property (Huang et al. 2021). However, this result was not reproducible in Arabidopsis (Kromdijk et al. 2020). Furthermore, tobacco transgenic plants expressing either *AtPIP1;2* or *AtPIP1;4* showed no increase in *g*_m_ or CO_2_ assimilation rate (Clarke et al. 2022). These inconsistencies may be attributed to the functional redundancy of PIP aquaporins and the absence of comprehensive studies based on structural biology for CO_2_ selectivity of aquaporins.

The mechanisms underlying CO_2_ selectivity in aquaporins remain poorly understood. Mori et al. (2014) investigated the CO_2_ permeability of five barley PIP2 aquaporins, revealing that the replacing the isoleucine positioned at the carboxyl end of the extracellular Loop E (between the transmembrane domains 5 and 6) with methionine abolished CO_2_ permeability while preserving water permeability. That finding highlighted the critical role of the Loop E in CO_2_ selectivity.

Aquaporins function as tetramers, with each monomer forming a water‐conducting pore (Kruse et al. 2006; Verkman et al. 1996). Two NPA boxes, each comprised of asparagine, proline and alanine, and an aromatic/arginine (ar/R) motif comprised of four amino acid residues, are postulated to function as water‐selective filters in the monomeric pores (Sui et al. 2001; Tajkhorshid et al. 2002). The tetrameric assembly is thought to create a central pore at the centre of the tetramer (Ozu et al. 2018). While the central pore has been proposed as a CO_2_ transport pathway in both animal (Hub and de Groot 2006) and plant aquaporins (Tyerman et al. 2021), direct experimental evidence remains lacking.

In this study, we examined the CO_2_ permeability of eight Arabidopsis PIP2 aquaporins utilizing the heterologous expression in *Xenopus laevis* oocytes and pinpointed critical amino acid residues involved in CO_2_ permeability by comparing CO_2_‐permeable and ‐nonpermeable PIP2 aquaporins to get insight into structural basis of CO_2_ selectivity of plant PIP2 aquaporins.

## 2 Materials and Methods

### 2.1 Plasmid Construction and Synthesis of Complementary RNA

cDNAs of *AtPIP2;1, 2;2, 2;3, 2;4, 2;5, 2;6, 2;7,* and *2;8* were subcloned into the pXβG‐ev1 expression plasmid vector using the unique *Bgl*II restriction site (Agre et al. 1993; Horie et al. 2011). Primer sequences used for cloning of the cDNA are listed in Supporting Information S1: Table S1. The plasmids were linearized with *Bam*HI (*AtPIP2;1, 2;3, 2;5* and *2;6*) or *Spe*I (*AtPIP2;2, 2;4, 2;7* and *2;8*), followed by the synthesis of cRNA using the mMessage mMachine T3 in vitro transcription kit (Ambion, Vilnius, Lithuania). Synthesis of single‐band cRNAs was checked by the denaturing RNA electrophoresis in an agarose gel.

Chimeric proteins of AtPIP2;1 and AtPIP2;3 were synthesized according to the method described elsewhere (Shibasaka et al. 2024). In brief, the donor and acceptor plasmids were mixed with four primers, and PCR was performed. Following *Dpn*I digestion to remove methylated template plasmid, the synthesized target plasmid was used for the transformation of *Escherichia coli* (strain DH5α) for subcloning. Primer sequences for each construction are shown in Supporting Information S1: Table S2.

PIP2;1 (N)‐PIP2;3(C), PIP2;3‐PIP2;1‐PIP2;3 and PIP2;1‐PIP2;3‐PIP2;1 chimeric proteins were constructed by the method described previously (Shibasaka et al. 2024). Single amino acid substitution series on PIP2;3‐PIP2;1‐PIP2;3: 3‐1‐3^Y150H^, 3‐1‐3^T152V^, 3‐1‐3^R153N^, 3‐1‐3^S160F^ and 3‐1‐3^S166N^ and amino acid substitution constructs: AtPIP2;1^Y150H^, AtPIP2;1^S160F^ and AtPIP2;3^H147Y‐F157S^ were constructed as described previously (Zheng 2004). The primers are listed in Supporting Information S1: Table S2.

### 2.2 Oocyte Swelling Assay

*P*_f_ of cRNA‐injected oocytes were examined by the swelling assay. Oocytes were isolated from adult female *X. laevis*, pretreated and injected with 50 nL of cRNA solution containing 10 or 25 ng of RNAs following a 24‐h incubation at 18°C in the modified Barth's solution (MBS) as previously described (Katsuhara et al. 2002) before the measurement of cell swelling. Water‐injected oocytes were used as the negative control. *HvPIP2;1* cRNA was used as a positive control (Mori et al. 2014). Measurement of increase in oocyte volume was carried out as previously described (Horie et al. 2011). The osmolality of MBS was changed from onefold MBS to 1/5‐fold MBS for the swelling initiation. Experiments involving *X. laevis* were conducted in accordance with the guidelines of the Institutional Animal Care and Use Committee of Okayama University under the approval number OKU‐2017271.

### 2.3 Micro pH Electrode Fabrication, Intracellular pH Measurement and *P*_CO2_ Calculation

Micro‐pH electrode for the impalement was fabricated as described previously (Mori et al. 2014). In brief, a glass capillary (TW150F, World Precision Instruments, Sarasota, Florida, USA) was pulled with a micropipette puller (MODEL P‐1000, Sutter Instrument, Novato, California, USA) to make a micropipette. The micropipette was backfilled with the mixture of protonophore cocktail (hydrogen ionophore I‐cocktail A, Sigma‐Aldrich, St. Louis, Missouri, USA), 0.5% polyvinyl chloride dissolved in tetrahydrofuran and tetrahydrofuran at 1:4:5 ratio following the hydrophobic treatment with tributyl chlorosilane. After an overnight solidification of the protonophore mixture, the micropipette was filled with a pH electrode solution (0.5 M KCl, 0.1 M Tris‐HCl, pH 7.0).

Oocytes were isolated from a *Xenopus laevis* female and injected with 25 ng/50 nL of cRNA solution and 25 ng carbonic anhydrase as described previously (Mori et al. 2014). After a 24‐h incubation at 18°C in MBS, the injected oocytes were subjected to the intracellular pH measurement. The pH in the oocyte was determined from the difference of voltage reading of micro‐pH electrode and membrane potential electrode using headstages, HS‐9A and HS‐2 (Molecular Devices, San Jose, California, USA), for membrane voltage and pH electrodes, respectively, and an amplifier (Axoclamp 900 A, Molecular Devices). Electric records were analysed with pClamp10 (Molecular Devices).

CO_2_ transport activity of AtPIP2s was determined by measuring the decrease in intracellular pH associated with the influx of carbon dioxide into the cell. The measurement was initiated by the replacement of the buffer from 11 µM to 6.5 mM at a perfusion rate of 0.4 mL s^−1^. The perfusion method and *P*_CO2_ were calculated by logarithmic curve fitting as previously described (Mori et al. 2014).

### 2.4 RNA Extraction and Qpcr

Total RNA was extracted from Arabidopsis mesophyll cell protoplast and guard cell‐enriched epidermis by Trizol reagent (Thermo Fisher Scientific Inc., Waltham, MA, USA) as previously reported (Mozhgani et al. 2024). cDNA was synthesized using Prime Script II reverse transcriptase (TaKaRa Bio Inc., Shiga, Japan), using 2.5 μg of total RNA and an oligo(dT) primer according to the manufacturer's instructions. PCR was performed with TB Green *Premix Ex* Taq II (Takara Bio Inc., Kusatsu, Japan) using LightCycler 96 Instrument (Roche, Basel, Switzerland). The primers for Actin and Arabidopsis PIP2 aquaporins were reported in an earlier study (Jang et al. 2004).

### 2.5 Phylogenetic Analysis

Amino acid sequences were collected from the Aramemnon website (https://aramemnon.botanik.uni-koeln.de/) and aligned with ClustalW with Neighbour Joining method using the full‐length amino acid sequences.

### 2.6 Immunoblotting and Immunohistochemistry

The crude membrane proteins were extracted from the *X. laevis* oocytes injected with 25 ng of *AtPIP2;1* or *AtPIP2;3* cRNAs after 24 h of incubation as previously described (Leduc‐Nadeau et al. 2007). Proteins from five oocytes were separated in a 10% polyacrylamide gel in the presence of sodium lauryl sulphate and transferred to a membrane sheet of polyvinylidene difluoride. After blocking with 5% skim milk and washing with phosphate‐buffered saline containing 0.1% Tween 20 (PBS‐T), the membrane was incubated for 1 h with the primary antiserum (anti‐aquaporin AtPIP2;1, AtPIP2;2, AtPIP2;3 antiserum COP‐080027, Cosmo Bio Co. Ltd., Tokyo Japan) in PBS‐T at a dilution factor of 1:1000 and a successive incubation with the secondary antiserum‐conjugated with horse radish peroxidase (anti‐rabbit IgG, horseradish peroxidase‐linked whole antibody from donkey, Cytiva, Marlborough, Massachusetts, USA) at a dilution factor of 1:10,000. Luminescence was visualized using the ECL chemiluminescence detection kit (Thermo Fisher Scientific, Waltham, Massachusetts, USA) and detected with a Chemiluminescence Imaging System (Fusion Solo S, Vilber Bio Imaging, Marne‐la‐Vallée, France). Band intensities were quantified using FIJI software (version 1.54 f) (Schindelin et al. 2012).

Immunohistochemistry was conducted according to the previous report (Shibasaka et al. 2021). Complementary RNA of each gene (25 ng per oocyte) was injected into *X. laevis* oocytes and incubated for 24 h at 18°C, followed by fixation, sliced with a microslicer (Zero 1, Dosaka EM Co. Ltd., Kyoto, Japan). Following the primary antibody treatment (anti‐aquaporin AtPIP2;1, AtPIP2;2, AtPIP2;3 antiserum COP‐080027, Cosmo Bio Co. Ltd., Tokyo, Japan), Alexa 488‐labelled secondary antibody treatment (Invitrogen, Waltham, MA, USA) was carried out as described previously (Shibasaka et al. 2021). Image of green fluorescence of Alexa Fluor‐488 was captured by a fluorescence microscope (BZ‐X700, Keyence Corporation, Osaka, Japan).

### 2.7 Three‐Dimensional Modelling by AlphaFold2

The tetramer structures of proteins (AtPIP2;1, AtPIP2;3, AtPIP2;1^Y150H^, AtPIP2;1^S160F^ and AtPIP2;3^H147Y‐F157S^) were predicted using AlphaFold‐multimer 2 (Mirdita et al. 2022). The model with the highest predicted local distance difference test score was used for the comparison. The model was visualized by Open‐Source PyMOL 3.0 (The PyMOL Molecular Graphics System, Schrödinger LLC).

## 3 Results

### 3.1 CO_2_ and Water Transport Activities of Arabidopsis PIP2 Aquaporins

**Figure 1:**
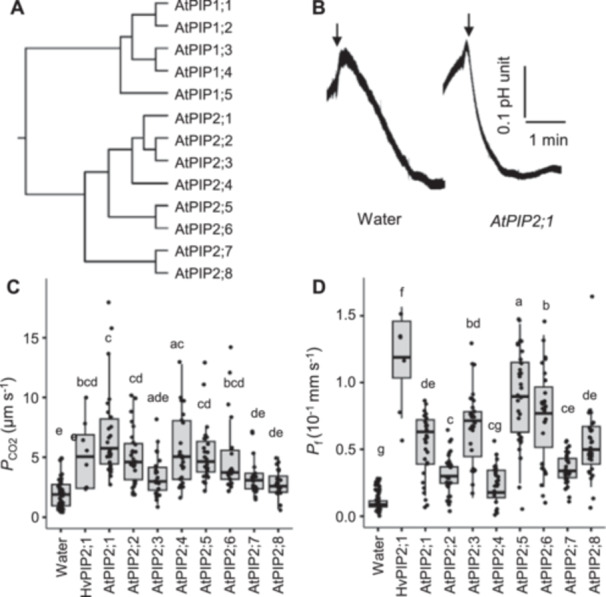
CO_2_‐and water‐permeability of Arabidopsis PIP2 aquaporins. (A) Phylogenetic tree of Arabidopsis PIP proteins. Phylogenetic tree was generated by ClustalW with Neighbour Joining method using the full‐length amino acid sequences. The Arabidopsis Gene Identifier codes are AtPIP1;1, At3g61430; AtPIP1;2, At2g45960; AtPIP1;3, At1g01620; AtPIP1;4, At4g00430; AtPIP1;5, At4g23400; AtPIP2;1, At3g53420; AtPIP2;2, At2g37170; AtPIP2;3, At2g37180; AtPIP2;4, At5g60660; AtPIP2;5, At3g54820; AtPIP2;6, At2g39010; AtPIP2;7, At4g35100; AtPIP2;8, At2g16850. (B) Raw recording of cytosolic pH of water and *AtPIP2;1* cRNA‐injected *X. laevis* oocytes by the micro‐pH electrode impalement method. Arrows indicate where the perfusion with 6.5 mM CO_2_/H_2_CO_3_‐containing solution initiated. (C) Diffusion coefficient of CO_2_ (*P*_CO2_) of the cell membrane of *X. laevis* oocytes injected with water (*n* = 44) or cRNAs of either *HvPIP2;1* (*n* = 8), *AtPIP2;1* (*n* = 25), *AtPIP2;2* (*n* = 27), *AtPIP2;3* (*n* = 23), *AtPIP2;4* (*n* = 23), *AtPIP2;5* (*n* = 24), *AtPIP2;6* (*n* = 22), *AtPIP2;7* (*n* = 22) or *AtPIP2;8* (*n* = 23). (D) Diffusion coefficient of water (*P*_f_) of the cell membrane of *X. laevis* oocytes injected with water (*n* = 56) or cRNA (10 ng/50 nL) of *HvPIP2;1* (*n* = 8), *AtPIP2;1* (*n* = 30), *AtPIP2;2* (*n* = 30), *AtPIP2;3* (*n* = 31), *AtPIP2;4* (*n* = 30), *AtPIP2;5* (*n* = 32), *AtPIP2;6* (*n* = 31), *AtPIP2;7* (*n* = 30) or *AtPIP2;8* (*n* = 29). *P*_f_ was measured by the oocyte swelling assay. Different letters show significant difference (*α* = 0.05) by Tukey–Kramer multiple comparison test.

The PIP aquaporin family in the Arabidopsis genome consists of 13 members, classified into PIP1 and PIP2 clades (Groszmann et al. 2023) (Figure 1A). To assess CO_2_ permeability of PIP2 aquaporins, we measured the diffusion coefficient of CO_2_ (*P*_CO2_) of the cell membrane in *X. laevis* oocytes injected with PIP2 complementary RNA (cRNA) using the micro‐pH electrode impalement method (Mori et al. 2014; Nakhoul et al. 1998). In *AtPIP2;1* cRNA‐injected oocytes, cytosolic pH decreased rapidly (with a 10 s‐lag) upon the onset of the perfusion with a high CO_2_ solution (6.5 mM), reaching the plateau faster than the water‐injected negative controls (Figure 1B). *AtPIP2;1, AtPIP2;2, AtPIP2;4, AtPIP2;5* and *AtPIP2;6* exhibited higher *P*_CO2_ values, comparable to the positive control (barley *HvPIP2;1* cRNA) (Horie et al. 2011; Mori et al. 2014) (Figure 1C). In contrast, *AtPIP2;3*, *AtPIP2;7* and *AtPIP2;8* showed low *P*_CO2_, indicating that five of eight PIP2 of Arabidopsis PIP2 facilitate CO_2_ transport, while the remaining three exhibit little to no CO_2_ permeability.

We also examined the diffusion coefficient of water (*P*_f_) using the oocyte swelling assay (Horie et al. 2011). All *AtPIP2* cRNA‐injected oocytes showed significantly higher *P*_f_ than the negative control, except *AtPIP2;4*, which exhibited low *P*_f_ (Figure 1D). These results suggest that AtPIP2;1, AtPIP2;2, AtPIP2;5 and AtPIP2;6 function as dual water‐ and CO_2_‐permeating aquaporins, whereas AtPIP2;3, AtPIP2;7 and AtPIP2;8 are primarily water‐selective. Notably, AtPIP2;4 appears to be CO_2_‐selective, with little water permeability. The low *P*_CO2_ in AtPIP2;3, AtPIP2;7 and AtPIP2;8 and low *P*_f_ in AtPIP2;4 were not attributed to failed translation and membrane integration, as all eight PIP2 proteins exhibited significant permeability to at least one substrate.

In contrast, *P*_f_ values of each *AtPIP1* cRNA‐injected oocytes were comparable to water‐injected controls (Supporting Information S1: Figure S1). Reportedly, the localization of most PIP1 aquaporins to the *X. laevis* oocyte cell membrane is insufficient (Mori et al. 2014; Shibasaka et al. 2021). Therefore, the low *P*_f_ observed for AtPIP1 aquaporins is likely due to their insufficient functional integration into the oocyte plasma membrane. Therefore, we focused our analysis on PIP2 aquaporins and did not further investigate PIP1 in this study.

### 3.2 Involvement of the Loop C Amino Acids in the CO_2_ Permeability of AtPIP2;1

AtPIP2;1 and AtPIP2;3 exhibited significantly different CO_2_‐permeability despite their sequence similarity (93.7% similarity and 90.9% identity) (Figure 1C and Supporting Information S1: Figure S2). To identify the critical amino acid residues responsible for this difference, we examined *P*_CO2_ in oocytes expressing chimeric proteins derived from AtPIP2;1 and AtPIP2;3. We focused on the N‐terminal franking region and loop C, since these regions had rich amino acid polymorphisms.

**Figure 2:**
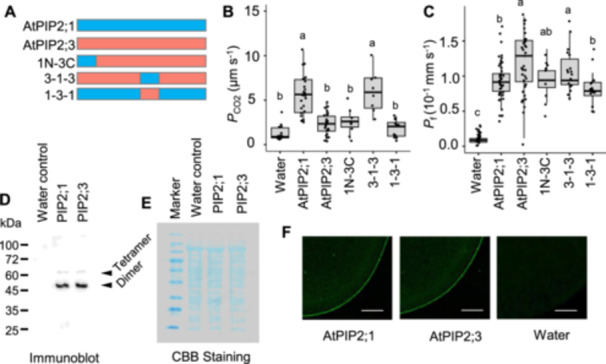
Involvement of loop C in the CO_2_ permeability of AtPIP2;1. (A) Schematic illustration of chimeric proteins. 1N‐3C, 3‐1‐3 and 1‐3‐1 indicate PIP2;1 (N)‐PIP2;3(C), PIP2;3‐PIP2;1‐PIP2;3 and PIP2;1‐PIP2;3‐PIP2;3, respectively. (B) Diffusion coefficient of CO_2_ (*P*_CO2_) of the cell membrane of *X. laevis* oocytes injected with water as the control (*n* = 17), and *AtPIP2;1* cRNA (*n* = 24), *AtPIP2;3* (*n* = 25), and the chimeric constructs [1N‐3C (*n* = 9), 3‐1‐3 (*n* = 8) and 1‐3‐1 (*n* = 11)]. (C) Diffusion coefficient of water (*P*_f_) of the cell membrane of *X. laevis* oocytes injected with water as control (*n* = 38), *AtPIP2;1* cRNA (*n* = 41), *AtPIP2;3* (*n* = 39) and the chimeric constructs, *1N‐3C* cRNA (*n* = 9), *3‐1‐3* cRNA (*n* = 21) and *1‐3‐1* cRNA (*n* = 17). Twenty‐five nanograms of cRNA were injected per oocyte for the oocyte swelling assay. Different letters indicate significant difference (*α* = 0.05) by the Tukey–Kramer multiple comparison test. (D) Immunoblot showing AtPIP2;1 and AtPIP2;3 protein accumulation in the crude membrane fraction of *X. laevis* oocytes. Crude membrane extract from five oocytes was loaded to a well. (E) Coomassie Brilliant Blue (CBB) staining serves as the loading control. Regular Range Protein Marker (PM1500, ExcelBAND, SMOBIO Technoloty Inc., New Taipei, Taiwan). Images of (D) and (E) are representative images from three biological replicates giving essentially the same results. (F) Localization of AtPIP2:1 and AtPIP2;3 in *X. laevis* oocytes injected with either water, *AtPIP2;1* cRNA or *AtPIP2;3* cRNA. A fluorescent image of a sliced PIP2;1, PIP2;3‐expressing oocyte and water‐injected oocytes. Exposure = 1 s. Sensitivity = +6 dB. Bar = 50 µm.

The PIP2;1 (N)‐PIP2;3(C) chimeric protein, which possessed the AtPIP2;1 N‐terminus with the AtPIP2;3 backbone, exhibited low *P*_CO2_ comparable to AtPIP2;3, indicating that the N‐terminus does not play a crucial role in CO_2_ permeability (Figure 2A,B). In contrast, the PIP2;3‐PIP2;1‐PIP2;3 chimera, containing the AtPIP2;1 loop C on the AtPIP2;3 backbone, displayed high *P*_CO2_ similar to AtPIP2;1, suggesting that loop C is sufficient to confer CO_2_ permeability. Consistently, PIP2;1‐PIP2;3‐PIP2;1, which introduced loop C of AtPIP2;3 into AtPIP2;1, resulted in low *P*_CO2_ (Figure 2B).

Unlike *P*_CO2_, *P*_f_ remained unchanged among AtPIP2;1, AtPIP2;3 and three chimeric proteins (Figure 2C), confirming that all proteins were functionally integrated into the oocyte cell membranes. Immunoblot and histochemical analyses further verified the successful expression and localization of AtPIP2;1 and AtPIP2;3. No nonspecific bands were detected in crude membrane extracts from water‐injected oocytes (Figure 2D,E). In oocytes injected with *AtPIP2;1* and *AtPIP2;3* cRNAs, two protein bands with similar immunoblot intensity were observed, corresponding to dimeric and tetrameric states (Figure 2D). Aquaporins exist as tetramers in vivo. During heating in SDS sample buffer, aquaporin molecules are expected to dissociate into monomers. However, under the standard SDS‐PAGE protocol, AtPIP2;1 and AtPIP2;3 predominantly retained a dimeric structure, with some molecules remaining as tetramers, and no visible monomeric band. Histochemical analysis confirmed their localization in the oocyte cell membranes (Figure 2F). These findings indicate that the low CO_2_ permeability of AtPIP2;3 was not caused by mislocalization but rather an intrinsic lack of CO_2_ permeability.

**Figure 3:**
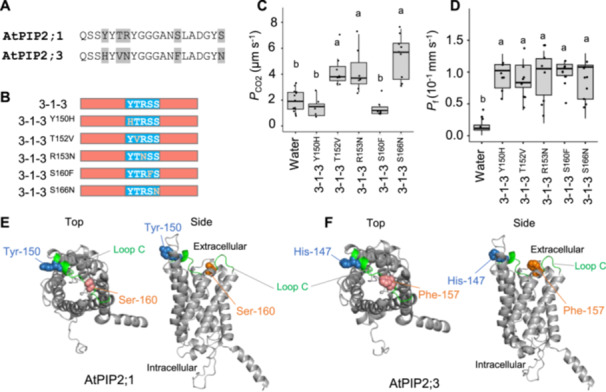
Exploration of the critical amino acid residues for determining CO_2_ permeability in the loop C. (A) Amino acid alignment of the loop C of AtPIP2;1 and AtPIP2;3. Residues highlighted with grey boxes indicate the dissimilar residues. (B) Schematic illustration of single amino acid substitution mutant proteins on the 3‐1‐3 backbone. (C) Diffusion coefficient of CO_2_ (*P*_CO2_) of the cell membrane of *X. laevis* oocytes injected with water (*n* = 13), single amino acid mutants, *3‐1‐3* ^*Y150H*^ cRNA (*n* = 5), *3‐1‐3* ^*T152V*^ cRNA (*n* = 8), *3‐1‐3* ^*R153N*^ cRNA (*n* = 7), *3‐1‐3* ^*S160F*^ cRNA (*n* = 7) and *3‐1‐3* ^*S166N*^ cRNA (*n* = 9). (D) Diffusion coefficient of water (*P*_f_) of the cell membrane of *X. laevis* oocytes injected with water (*n* = 7), single amino acid mutants, *3‐1‐3* ^*Y150H*^ cRNA (*n* = 7), *3‐1‐3* ^*T152V*^ cRNA (*n* = 8), *3‐1‐3* ^*R153N*^ cRNA (*n* = 8), *3‐1‐3* ^*S160F*^ cRNA (*n* = 8) and *3‐1‐3* ^*S166N*^ cRNA (*n* = 9). Twenty‐five nanograms of cRNA were injected per oocyte for the oocyte swelling assay. Three‐dimensional structures of AtPIP2;1 (E) and AtPIP2;3 (F) modelled by AlphaFold2 multimer2. Only a monomer is visualized. The loop C is shown in green. The amino acid residues involved in the difference in CO_2_‐permeability between AtPIP2;1 and AtPIP2;3: tyrosine‐150/histidine‐147 and serine‐160 and phenylalanine‐157 are illustrated by blue and orange spheres, respectively.

To pinpoint key residues in loop C affecting CO_2_ permeability, we examined *P*_CO2_ in a series of single amino acid substitutions in the PIP2;3‐PIP2;1‐PIP2;3 chimeric protein. This region included five amino acid polymorphisms (Figure 3A,B). Substituting threonine‐152 with valine, arginine‐153 with asparagine, or serine‐166 with asparagine had no effect on CO_2_ permeability (Figure 3C). However, replacing tyrosine‐150 with histidine or serine‐160 with phenylalanine abolished the CO_2_ permeability, while *P*_f_ remained unaffected among all mutants (Figure 3C,D). These results suggest that the reduced CO_2_ permeability of AtPIP2;3 compared with AtPIP2;1 is chiefly due to the tyrosine/histidine and serine/phenylalanine polymorphisms in loop C, which is positioned near the extracellular face of the monomeric pore (Figure 3E,F).

**Figure 4:**
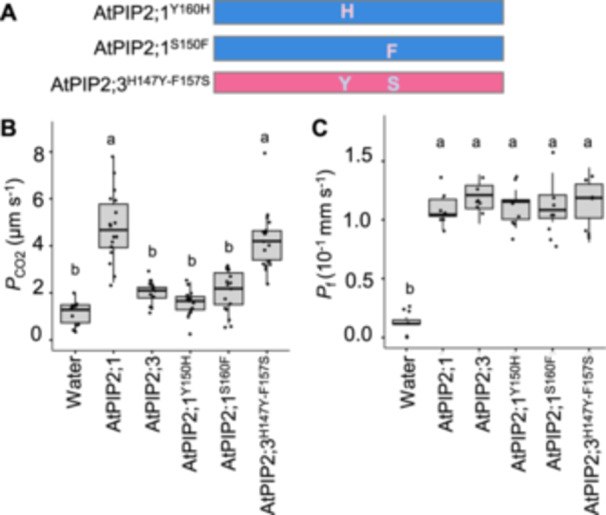
Tyrosine and serine residues in the loop C are critical for determining CO_2_ selectivity in AtPIP2;1. (A) Schematic illustration of amino acid substitution in AtPIP2;1 and AtPIP2;3. (B) Diffusion coefficient of water (*P*_CO2_) of the cell membrane of *X. laevis* oocytes injected with water (*n* = 12), *AtPIP2;1* cRNA (*n* = 18), *AtPIP2;3* cRNA (*n* = 14), and the amino acid‐substituted constructs, *AtPIP2;1* ^*Y150H*^ cRNA (*n* = 15), *AtPIP2;1* ^*S160F*^ cRNA (*n* = 16) and *AtPIP2;3* ^*H147Y‐F157S*^ cRNA (*n* = 17). (C) Diffusion coefficient of water (*P*_f_) of the cell membrane of *X. laevis* oocytes injected with water (*n* = 6), *AtPIP2;1* cRNA (*n* = 7), *AtPIP2;3* cRNA (*n* = 7), *AtPIP2;1* ^*Y150H*^ cRNA (*n* = 7), *AtPIP2;1* ^*S160F*^ cRNA (*n* = 8) and *AtPIP2;3* ^*H147Y‐F157S*^ cRNA (*n* = 8). Twenty‐five nanograms of cRNA were injected per oocyte for the oocyte swelling assay. Different letters indicate significant difference (*α* = 0.05) by Tukey–Kramer multiple comparison test.

To further validate this, we generated three single amino acid substitution constructs: AtPIP2;1 protein that possessed single amino acid substitutions, AtPIP2;1^Y150H^ and AtPIP2;1^S160F^, and AtPIP2;3 protein that possessed a two amino‐acid‐substitution AtPIP2;3^H147Y‐F157S^ (Figure 4A). Examining *P*_CO2_ revealed that substituting tyrosine‐150 with histidine or serine‐160 with phenylalanine in AtPIP2;1 abolished CO_2_ permeability (Figure 4B). Conversely, replacement of two amino acid residues in AtPIP2;3, histidine‐147 to tyrosine and phenyalanine‐157 to serine, conferred CO_2_ permeability (Figure 4B). Since *P*_f_ remained comparable among all constructs (Figure 4C), variability of CO_2_ permeability was not due to translation efficiency or mislocalization but the effect of these residues on the activity.

Together, our findings strongly suggest that tyrosine‐150 and serine‐160 in loop C are critical for CO_2_ selectivity in PIP2 aquaporins.

### 3.3 AlphaFold‐Based Structural Modelling and Sidechain Conformation Change

**Figure 5:**
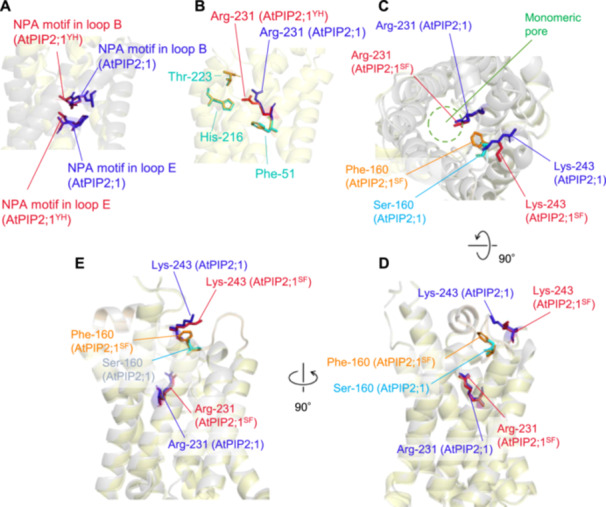
Conformational change of sidechains between AtPIP2;1, AtPIP2;1^Y150H^ and AtPIP2;1^S160F^ modelled by AlphaFold2. (A) NPA boxes are shown by sticks. Blue and red sticks indicate AtPIP2;1 and AtPIP2;1^Y150H^, respectively. (B) Four amino acid residues comprising the ar/R motif. Phenylalanine‐51, histidine‐216 and threonine‐223 are indicated by turquoise and orange in AtPIP2;1 and AtPIP2;1^Y150H^, respectively. Arginine‐231 is indicated by blue and red, respectively. AtPIP2;1^Y160H^ is designated as AtPIP2;1^YH^. (C–E) The serine and phenylalanine residues that are substituted in AtPIP2;1 and AtPIP2;1^S160F^ are shown by turquoise and orange, respectively. Arginine‐231 and lysine‐243 are shown in blue (AtPIP2;1) and red (AtPIP2;1^S160F^). The main peptide chains of AtPIP2;1 are shown in grey. The main peptide chains of AtPIP2;1^Y150H^ and AtPIP2;1^S160F^ are shown in pale yellow.

The three‐dimensional structures of AtPIP2;1, AtPIP2;3, AtPIP2;1^Y150H^, AtPIP2;1^S160F^ and AtPIP2;3^H147Y‐F157S^ were modelled using AlphaFold2 to predict the conformational changes induced by critical amino acid substitutions (Figure 5 and Supporting Information S1: Figure S3).

Structural comparison of AtPIP2;1 and AtPIP2;3 revealed no apparent difference in the position of α‐carbons across the entire proteins (Figure 3E,F). The NPA box sidechains were also structurally conserved between AtPIP2;1, AtPIP2;3 and AtPIP2;1^Y150H^ (Figure 5A). However, a notable conformational difference was observed in arginine‐231 between AtPIP2;1 and AtPIP2;1^Y150H^. In AtPIP2;1, the arginine‐231 sidechain aligned parallel to the monomeric pore axis, whereas in AtPIP2;1^Y150H^, it was oriented transversely (Figure 5B). This arginine, one of the residues comprising the ar/R motif (Inden et al. 2023), function as a water selection filter alongside the NPA boxes. Its transverse orientation may obstruct CO_2_ permeation through the monomeric pore. Notably, a similar transverse orientation was observed in the CO_2_‐impermeable AtPIP2;3 (Supporting Information S1: Figure S3). Conversely, in the CO_2_‐permeable AtPIP2;3^H147Y‐F157S^, the conserved arginine sidechain was repositioned parallel to the pore axis, suggesting a role for arginine‐231 conformation in CO_2_ selectivity.

Further conformational changes were found in AtPIP2;1^S160F^. In this mutant, phenylalamine‐160 extended over the monomeric pore more remarkably than serine in AtPIP2;1 (Figure 5C). This bulky hydrophobic moiety appeared to displace lysine‐243 outward, away from the tetrameric aquaporin complex (Figure 5C–E). Additionally, the substitution from serine to phenylalanine caused a slight shift (0.9 Å) of arginine‐231 within the ar/R motif toward the interior of the monomeric pore (Figure 5C–E). Though modest, this shift may alter the electric charge distribution in the ar/R constriction within the monomeric pore, potentially hampering the access of the hydrophobic CO_2_ molecule to the pore interior.

## 4 Discussion

Earlier studies have shown that certain aquaporins facilitate CO_2_ transport across biomembranes. Human AQP1 and AQP5 have been reported to transport CO_2_ in *X. laevis* oocyte and proteoliposomes (Alishahi and Kamali 2019; Musa‐Aziz et al. 2009; Geyer et al. 2013). Additionally, CO_2_ permeability has been observed in bovine AQP0, rat AQP5, AQP6 and AQP9, as well as zebrafish AQP1a1 (Musa‐Aziz et al. 2009; Geyer et al. 2013; Talbot et al. 2015). Aquaporins of the cyanobacterium (*Synechococcus* sp. PCC7942) and marine diatoms (*Phaeodactylum tricornutum* and *Thalassiosira pseudonana*) have also been implicated in CO_2_ transport (Ding et al. 2013; Matsui et al. 2018).

In terrestrial plants, NtAQP1, a PIP1 aquaporin from tobacco, was shown to facilitate CO_2_ permeation when expressed heterologously in *Xenopus* oocytes (Uehlein et al. 2003). The report regarding tobacco NtPIP2;1 is complicated; no apparent CO_2_ permeability was found when expressed in the yeast cell‐heterologous expression system (Otto et al. 2010), but observed using polymer‐embedding assay (Kai and Kaldenhoff 2014; Uehlein, Otto, et al. 2012). Arabidopsis PIP1;2 was reported to permeate CO_2_, whereas AtPIP2;3 did not (Heckwolf et al. 2011). Other studies identified CO_2_ permeability in AtPIP2;1 and AtPIP2;5 (Israel et al. 2021; C. Wang et al. 2016), while the CO_2_ transport capacities of AtPIP2;2, AtPIP2;4, AtPIP2;6, AtPIP2;7 and AtPIP2;8 remained untested. Comprehensive analyses in barley (*Hordeum vulgare*) and foxtail millet (*Setaria italica*) have further highlighted variation in CO_2_ permeability in PIP2 aquaporins. Four out of five barley PIP2 isoforms (HvPIP2;1, HvPIP2;2, HvPIP2;3 and HvPIP2;5) exhibited significant CO_2_ permeability, while HvPIP2;4 did not (Mori et al. 2014). In foxtail millet, only SiPIP2;7 was CO_2_ permeable, whereas SiPIP2;1, SiPIP2;4 and SiPIP2;5 were impermeable (Ermakova et al. 2021).

In this study, we comprehensively examined the CO_2_ permeability of Arabidopsis PIP2 aquaporins, revealing novel insight into their CO_2_ and water transport properties. Our findings confirmed previous reports of CO_2_ permeability in AtPIP2;1 and AtPIP2;5 (Israel et al. 2021) and reproduced the impermeability of AtPIP2;3 (Heckwolf et al. 2011). Additionally, we found that AtPIP2;7 and AtPIP2;8 lacked CO_2_ permeability. Notably, AtPIP2;4 exhibited CO_2_ permeability despite having significantly lower water permeability. These results underscore substantial differences in CO_2_ transport activity among plant PIP aquaporin isoforms.

In this study, we investigated the structural basis of CO_2_ permeability in PIP2 aquaporins. Despite earlier studies on aquaporin‐mediated CO_2_ transport, predicting CO_2_ permeability from primary structure remains challenging. A previous study identified a critical isoleucine in loop E as essential for CO_2_ permeation in barley HvPIP2;3 (Mori et al. 2014). Substituting this isoleucine with methionine introduced a nucleophilic interaction between the sulphur atom in the methionine and the oxygen atom in loop C, rendering the aquaporin CO_2_‐impermeable. To our knowledge, this is the only report structural determinant of aquaporin CO_2_ selectivity. In Arabidopsis, this isoleucine residue is strictly conserved among all eight PIP2 aquaporins (Mori et al. 2014). Thus, it does not explain the lack of CO_2_ permeability in AtPIP2;3, AtPIP2;7 and AtPIP2;8, suggesting that other amino acids play a role in determining CO_2_ permeability. To address this, we analysed chimeric proteins and single amino acid substitution between the CO_2_‐permeable AtPIP2;1 and the non‐/low‐permeable AtPIP2;3. Our results identified two critical amino acid polymorphisms responsible for CO_2_ transport. AlphFold2 modelling further revealed that these amino acid substitutions induce conformational changes in the arginine residue of the ar/R motif and/or hydrophobicity profile at the extracellular pore mouth. These structural changes likely account for the observed difference in CO_2_ permeability between AtPIP2;1 and AtPIP2;3.

Aquaporins form a tetrameric structure, with each monomer containing a narrow pore lined with conserved amino acids that selectively regulate molecular passage. It is well established that water molecules traverse the monomeric pore, where the NPA boxes and ar/R motif function as substrate‐selective filters (Kapilan et al. 2018; Lee et al. 2005; Sui et al. 2001). However, the pathway for CO_2_ transport remains unresolved—where it occurs through the monomeric pore or the central pore? While some studies have proposed the central pore as the primary CO_2_ passage way, concrete experimental evidence is lacking (Hub and de Groot 2006; Tyerman et al. 2021). Our findings support the monomeric pore as the likely route for CO_2_ permeation. We identified two key residues in AtPIP2;1, tyrosine‐150 and serine‐160, located in loop C, as critical for CO_2_ permeability (Figure 3A–C). Substituting tyrosine‐150 with histidine induced conformational change of arginine‐231 of the ar/R motif, a key residue in the monomeric pore (Figure 5). Similarly, replacing serine‐160 with phenylalanine altered the conformation of lysine‐243 and arginine‐231. Both phenylalanine‐160 and lysine‐243 (phenylalanine‐157 and lysine‐240 in AtPIP2;3, respectively) were positioned near the extracellular opening of the monomeric pore. Notably, these structural shifts were observed exclusively in proximity to the monomeric pore, rather than the central pore.

The tyrosine residue in loop C is only partially conserved among PIP2 aquaporins (Supporting Information S1: Figure S4). Among other CO_2_‐permeable aquaporins, barley HvPIP2;2 and HvPIP2;3 possess alanine and phenylalanine at this position, while foxtail millet SiPIP2;7 has glutamine. Variability is also observed at the serine position in loop C. It can be threonine, glutamic acid, glycine or methionine in CO_2_‐permeable aquaporins (Supporting Information S1: Figure S4).

Substituting tyrosine in loop C with histidine induced a remote conformational change in the arginine of the ar/R filter (Figure 5A,B and Supporting Information S1: Figure S3). However, no similar change was observed for the previously identified isoleucine in loop E of barley PIP2 aquaporins. Despite their similar primary structures, HvPIP2;3 and HvPIP2;4 exhibit different CO_2_ permeability (Mori et al. 2014). Structural modelling using AlphaFold2 predicted that arginine‐235 adopts an upright conformation in both HvPIP2;3 and HvPIP2;4 (Supporting Information S1: Figure S5), suggesting that the mechanisms regulating CO_2_ transport differ between AtPIP2;1/AtPIP2;3 and HvPIP2;3/HvPIP2;4.

We envisage that both CO_2_ and water molecules pass through the same proteinaceous pore. Here, we discuss a hypothetical mechanism by which the same pore accommodates both the hydrophilic water molecule and the hydrophobic CO_2_ molecule. Substrate selectivity is thought to depend on the interplay between solute properties, pore size and polarity (Kitchen et al. 2019). With regard to size, the diameter of the ar/R filter constriction is estimated to be approximately 3 Å (Murata et al. 2000). The estimated diameters of a water molecule, based on bond length and van der Waals radii, are approximately 3.3 Å (short axis) and 3.9 Å (long axis), while those of a CO_2_ molecule are approximately 3.4 and 5.4 Å, respectively. Both molecules are slightly larger than the pore diameter along their long axes, but their short axes are close to or slightly exceed the estimated constriction size. However, substrates may still pass through the ar/R filter via an induced fit mechanism, enabled by minor conformational changes and various noncovalent interactions. Thus, from a size perspective, both water and CO_2_ are considered permeable through the pore. The ar/R filter excludes ions primarily due to their charge and the large size of their hydration shells (de Groot and Grubmüller 2005; Murata et al. 2000; Wallace and Roberts 2004). Water, being a small polar molecule, passes readily through the filter. CO_2_, in contrast, is a linear and nonpolar molecule under isolated conditions. However, in polar environments, such as aqueous solutions or in close proximity to polar amino acid residues, CO_2_ can develop an induced dipole through transient intramolecular polarization. In this state, electron density shifts toward the oxygen atoms, while that around the central carbon decreases. This temporary dipole may facilitate the passage of CO_2_ through the ar/R filter despite its intrinsic non‐polarity. Pore shape is also likely to influence substrate permeability. In AtPIP2;3 and AtPIP2;1^Y150H^, the arginine residue in the ar/R filter adopts a transverse orientation relative to the pore axis. This leads to a bent pore geometry that may hinder the passage of the long, ellipsoidal CO_2_ molecule, while still permitting the relatively round‐shaped water molecule. In contrast, aquaporins with a parallel arginine orientation, such as AtPIP2;1 and AtPIP2;3^H147Y‐F157S^, appear to form a straighter pore structure, which may allow both water and CO_2_ to permeate without structural hindrance.

Phylogenetic analysis indicates that aquaporins capable of transporting both CO_2_ and water are widely distributed across clades within the PIP2 subfamily (Supporting Information S1: Figure S6). H_2_O‐selective and CO_2_‐selective aquaporins emerged independently in multiple clades, suggesting that dual‐permeating aquaporins served as evolutionary ancestors from which specialized forms later emerged (Supporting Information S1: Figure S6). As discussed above, the mechanisms restricting CO_2_ transport in single‐selective aquaporins likely vary among PIP2 isoforms. If we assume that dual‐permeating aquaporins represent the ancestral state and single‐selective variants emerged more recently, the diverse mechanisms underlying CO₂ impermeability can be reasonably explained within this evolutionary context.

The Arabidopsis genome has eight PIP2 aquaporins. In this study, we identify three aquaporins, AtPIP2;3, AtPIP2;7 and AtPIP2;8 as CO_2_‐impermeable (Figure 1) and pinpoint the key amino acids responsible for the lack of CO_2_ permeability in AtPIP2;3 (Figure 4). However, the molecular basis for CO_2_ impermeability in AtPIP2;7 and AtPIP2;8 remains unclear. Additionally, we found that AtPIP2;4 selectively permeates CO_2_ but not water, a unique characteristic whose underlying structural determinants were not explored in this study.

CO_2_‐permeable aquaporins have also been identified in vertebrates (Musa‐Aziz et al. 2009; Geyer et al. 2013; Talbot et al. 2015). To determine whether a common structural feature governs CO_2_ permeability across different kingdoms, we compared the primary structures of loop C in animals, plants and cyanobacterial aquaporins. However, loop C sequences exhibited high divergence, with no apparent conservation across taxa (Supporting Information S1: Figure S7). This suggests that the structural adaptations enabling CO_2_ transport in aquaporins are highly diverse and have evolved independently in different evolutionary lineages.

PIP aquaporins have been implicated in the regulation of *g*_m_ (Ermakova et al. 2021; Hanba et al. 2004; Heckwolf et al. 2011; Kawase et al. 2013; Terashima and Ono 2002; Uehlein et al. 2003; Uehlein, Sperling, et al. 2012; Xu et al. 2019). However, contradictive results have also been reported (Clarke et al. 2022; Kromdijk et al. 2020). To clarify the role of PIP aquaporins in *g*_m_, it is essential to identify CO_2_‐permeable aquaporins and characterize their expression in mesophyll cells. In addition to the role in *g*_m_, they are implicated to function in CO_2_/HCO_3_^−^ sensing by coupling with plasma membrane‐associated carbonic anhydrase (Hu et al. 2010). To assess the potential involvement of Arabidopsis PIP2 aquaporins in *g*_m_ and CO_2_/HCO_3_^−^ sensing in guard cells, we conducted quantitative reverse‐transcription PCR using total RNA extracted from the isolated mesophyll protoplasts, the guard cell‐enriched epidermal preparation and the rosettes (Supporting Information S1: Figure S8). The transcript levels of CO_2_‐permeable aquaporins, *AtPIP2;1*, *AtPIP2;2* and *AtPIP2;5* in the mesophyll protoplasts were comparable to those in the whole rosette leaves. However, *AtPIP2;4*, which also exhibits CO_2_ permeability, was significantly more abundant in mesophyll cells, suggesting a potential role in maintaining sufficiently high *g*_m_ to support photosynthesis. In contrast, *AtPIP2;6* expression was lower in mesophyll cells than in whole rosette, implying a lesser contribution to *g*_m_. PIP2 aquaporins that lacked substantial CO_2_ permeability are unlikely to play a major role in *g*_m_. In this study, we did not assess the phenotypes of knockout mutants for ‘COOporins’ in mesophyll cells. Disrupting multiple aquaporins could interfere water transport, potentially causing pleiotropic effects. Additionally, the CO_2_ permeability of PIP1 aquaporins remains unexplored, further complicating functional analysis. Future studies should address these gaps to clarify the precise role of aquaporin in *g*_m_ regulation.

## 5 Conclusion

We found that the tyrosine and serine residues in the loop C of AtPIP2;1 are important for CO_2_ transport, when the protein was expressed in *X. laevis* oocytes. Swapping the amino acids to those of the no/low CO_2_‐permeable AtPIP2;3 caused loss of CO_2_ transport activity. AlphaFold2 modelling suggested that the amino acid swapping effects on the conformation of distal amino acid residues adjacent to the monomeric pore of the aquaporin structure, rather than the central pore, suggesting CO_2_ permeates through the monomeric pore.

## Supporting information

Supporting material.

## Data Availability

The data that support the findings of this study are available from the corresponding author upon reasonable request.
