# Supplementary material for: Amino Acid Substitutions in Loop C of Arabidopsis PIP2 Aquaporins Alters the Permeability of CO2
Source: Plant Cell Environ. 2025 Jun 3;48(9):6835–46. doi: 10.1111/pce.15635 (PMC12319296; doi:10.1111/pce.15635)
Supplement: Supplementary file 1 — Supporting material. [file PCE-48-6835-s001.docx]

Supporting Information for

**Amino acid substitutions in loop C of PIP2 aquaporins alters the permeability of**

**CO_2_ in Arabidopsis.**

Shaila Shermin Tania, Shigeko Utsugi, Yoshiyuki Tsuchiya, Shizuka Sasano, Maki

Katsuhara, and Izumi C Mori

Izumi C. Mori

E-mail: [imori@okayama-u.ac.jp](mailto:imori@okayama-u.ac.jp)

**This PDF file includes:** Supplemental method Tables S1 to S2

Supplementary results: Figures S1 to S8

**Supplemental method**

**Calculation of *P*f**

The rate of water transport into the oocytes were determined from the initial rate of inflation of the volume of the oocytes. The projected images of oocytes were captured using an inverted microscope equipped a charged-coupled device camera (Cool SNAP, Photometrics, Tucson, AZ, USA). The projected areas of oocytes were measured by ImageJ. Capturing the images were carried out from 0 to 90 seconds immediately after a quick perfusion of the bathing solution with 10-second intervals.

*P*f was calculated from the measured projected areas by the equation (1).

𝑃 = 𝑉_𝑡_

(1)

𝑓

𝑆_0_

⋅ 𝑉_𝖶_

⋅ Δ𝑂𝑠𝑚 ⋅ 𝑡

*V*t is volume of the oocyte at time *t*, *S*0 is initial surface area of the oocyte, *V*w is the volume of water. ΔOsm is the difference in the osmolality. *t* is time from start of the measurement. *V*t and *S*0 are obtained as equations (2, 3)

𝑉_𝑡_ =

3

/ 𝑡

𝐴

4

3 π

(2)

𝑆_0_ = 4 ⋅ 𝐴_0_ (3)

*A*t is projected area of oocyte at time t, *A*0 is the initial projected area of oocyte.

**Calculation of *P*CO2**

The pH changes in oocytes by perfusing high CO2 solution showed an apparent logarithmic decrease curve (Mori et al. 2014; Nakhoul et al. 1998; Figure 1B). *P*CO2 was calculated from the time constant (*τ*) of the logarithmic curve of pH decrement according to the equation (4).

𝑃 1 (𝑝𝐻𝑓−𝑝𝐾𝑎) ⋅ 𝑉

(4)

_𝐶02_ = τ ⋅ 10 𝑆

pHf is final pH of the oocyte, p*K*a is p*K*a of carbonic acid, *V* is volume of oocyte, *S* is surface area of oocyte.

**Table S1**. Primer sequences used for cloning of cDNA

| Primers | Nucleotide sequence (5’ to 3’) |
| --- | --- |
| AtPIP2;1-F | GGAGATCTATGGCAAAGGATGTGGAAGC |
| AtPIP2;1-R | CCGCATGCTTAGACGTTGGCAGC |
| AtPIP2;2-F | ATGGCCAAAGACGTGGAAGGACCT |
| AtPIP2;2-R | TCAAACGTTGGCTGCACTTCTGAA |
| AtPIP2;3-F | GGAGATCTACAATGGCTAAAGACGTGG |
| AtPIP2;3-R | CCGCATGCTTAAACGTTGGCTGACTTC |
| AtPIP2;4-F | GGGCATGCACAATGGCAAAAGACTTGGATG |
| AtPIP2;4-R | CCGATATCTTAAGCAAAGC TCCTAAAGG |
| AtPIP2;5-F | GGGCAGCACAATGACGAAGGAAGTGG |
| AtPIP2;5-R | CCGATATCTTAAACGTGAGGCTGGCTC |
| AtPIP2;6-F | GGAGATCTAGAATGACGAAGGATGAGTTGACGG |
| AtPIP2;6-R | CCGCATGCTTAAGCATGGAGCTCATGAAGC |
| AtPIP2;7-F | GGAGATCTACAATGTCGAAAGAAGTGAGCG |
| AtPIP2;7-R | CCGCATGCTTAATTGGTTCCCTTGCTTC |
| AtPIP2;8-F | GGAGATCTACAATGTCAAAAGAACTCAGTGAAG |
| AtPIP2;8-R | CCGCATGCTCAATTGGTTGGGTTGCTG |

**Table S2**. Primer sequences for chimeric constructs and amino acid substitutions

| PIP2;1(N)-PIP2;3(C)  Insert forward (#1)  Insert reverse (#2)  Out forward (#3)  Out reverse (#4) | TGGCAGAACCATGGCAAAGGATGTGGAAGC GGCGATGACTGCTCTGTAGAAAGACCACTTCT GTGGTCTTTCTACAGAGCAGTCATCGCC  ATCCTTTGCCATGGTTCTGCCAAAGTTGAG |
| --- | --- |
| PIP2;3-PIP2;1-PIP2;3  Insert forward (#1)  Insert reverse (#2)  Out forward (#3)  Out reverse (#4) | GCATTTCAAAGCTCTTACTACACCCG GGTGCCTGTGCTGTAGCCATCG GATGGCTACAATACAGGCACCGGA  GTAGTAAGAGCTTTGAAATGCCTTCACAAAC |
| PIP2;1-PIP2;3-PIP2;1  Insert forward (#1)  Insert reverse (#2)  Out forward (#3)  Out reverse (#4) | AAGCCTTCCAAAGCTCTCACTATGTTAATTACGGT CGGTCCCTGTATTGTAGCCGTCAGCTAGAA CGGCTACAATACAGGGACCGGTCTA  AGTGAGAGCTTTGGAAGGCTTTGACAAAA |
| AtPIP313Y150H  Forward  Reverse | AAAGCTCTCACTACACCCGTTAC  CGGGTGTAGTGAGAGCTTTGAA |
| AtPIP313T152V  Forward  Reverse | CTCTTACTACGTTCGTTACGGAGGTGGAG  CCTCCGTAACGAACGTAGTAAGAGCTTTG |
| AtPIP313R153N  Forward  Reverse | CTTACTACACCAATTACGGAGGTGGAGC  CACCTCCGTAATTGGTGTAGTAAGAGC |
| AtPIP313S160F  Forward  Reverse | GGAGCCAACTTTCTAGCCGATGGC  CATCGGCTAGAAAGTTGGCTCCACC |
| AtPIP313S166N  Forward  Reverse | CGATGGCTACAATACAGGCACCGGACTC  CGGTGCCTGTATTGTAGCCATCGGCTAG |
| AtPIP2;1Y150H  Forward  Reverse | AAAGCTCTCACTACACCCGTTAC  CGGGTGTAGTGAGAGCTTTGAA |
| AtPIP2;1S160F  Forward  Reverse | GGAGCCAACTTTCTAGCCGATGGC  CATCGGCTAGAAAGTTGGCTCCACC |
| AtPIP2;3H147Y-F157S  AtPIP2;3HY forward AtPIP2;3HY reverse AtPIP2;3FS forward  AtPIP2;3FS reverse | CAAAGCTCTTACTATGTTAATTACG ATTAACATAGTAAGAGCTTTGAAATG GAGCCAACTCTCTAGCTGACGG  TCAGCTAGAGAGTTGGCTCCTCC |

**Supplementary results**

**Figure S1.** Water permeability of PIP1 aquporins of Arabidopsis. Diffusion coefficient of water (*P*_f_) of the cell membrane of *X. laevis* oocytes injected with water (n = 13), *AtPIP2;1* cRNA (n = 6), *AtPIP1;1* cRNA (n = 9), *AtPIP1;2* cRNA (n = 9), *AtPIP1;3* cRNA (n = 8), *AtPIP1;4* cRNA (n = 9)

and *AtPIP1;5* cRNA (n = 7). 25 ng cRNA was injected per oocyte. Different letters show significant difference (α = 0.05) by the Tukey- Kramer multiple comparison test. AtPIP2;1 serves as the positive control.

1.5

1.0


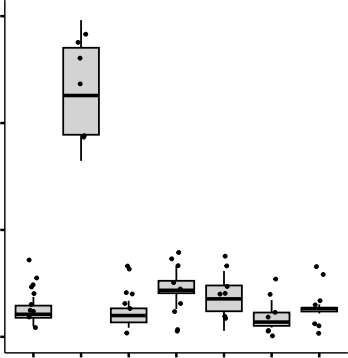


a

bc

b

bc

bc

c

bc

*P*_f_ (10-1 mmS-1)

0.5

0.0

Water

AtPIP2;1

AtPIP1;1

AtPIP1;2

AtPIP1;3

AtPIP1;4

AtPIP1;5

**AtPIP2;1**

**AtPIP2;3**

**AtPIP2;1**

**AtPIP2;3**

N-terminus

TM1

**AtPIP2;1 AtPIP2;3**


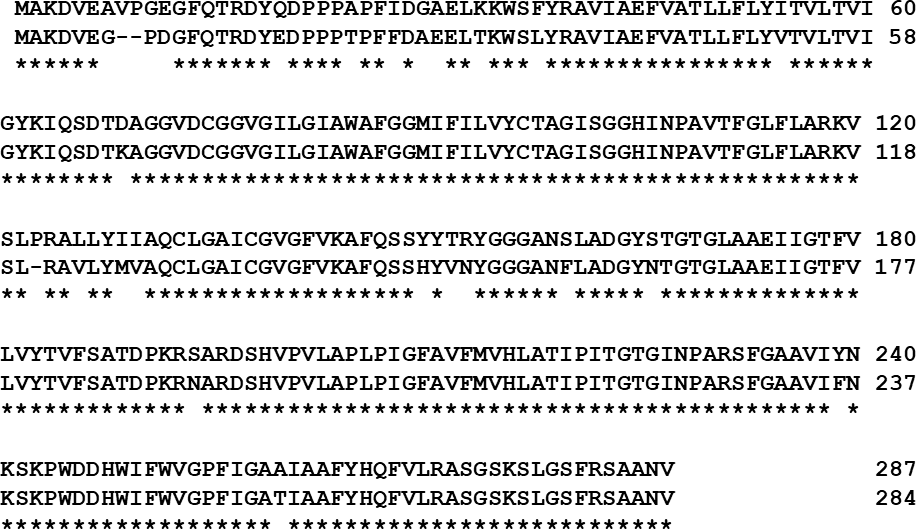


Loop A

TM2

Loop B

TM3

Loop C

TM4

Loop D

TM5

Loop E

TM6

C-terminus

**AtPIP2;1**

**AtPIP2;3**

**AtPIP2;1**

**AtPIP2;3**

**Figure S2.** Sequence alignment of apparent closer positioning AtPIP2;1 and AtPIP2;3 with distinct CO_2_ permeability. Similarity 93.73% and Identity percentage is 90.94%.The red marked boxes indicating N-terminus and Loop C shows apparent higher polymorphism parts. The transmembrane domain and loops were predicted by TMHMM software (https://services.healthtech.dtu.dk/services/TMHMM-2.0/).

ar/R motif

AtPIP2;1 AtPIP2;3 AtPIP2;3^H147Y-F157S^

NPA box


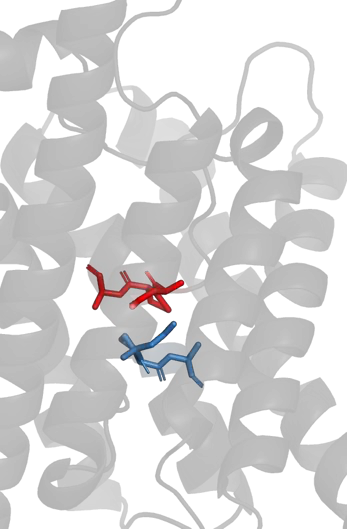


**NPA box in loop E**

**NPA box in loop B**


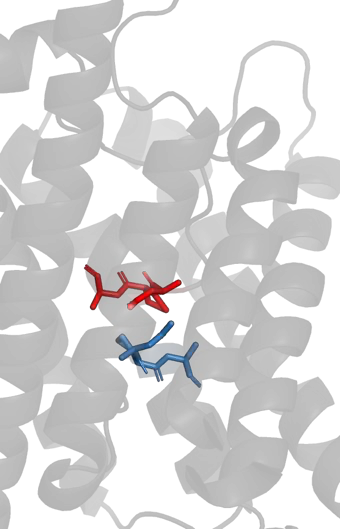


**NPA box in loop E**

**NPA box in loop B**


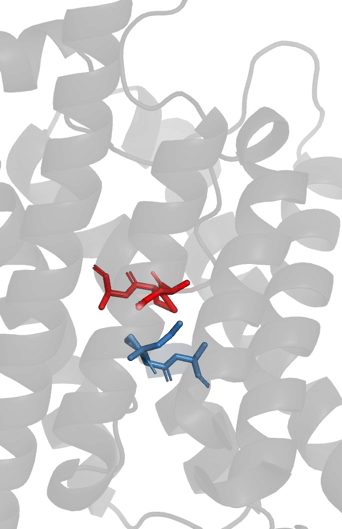


**NPA box in loop E**

**NPA box in loop B**


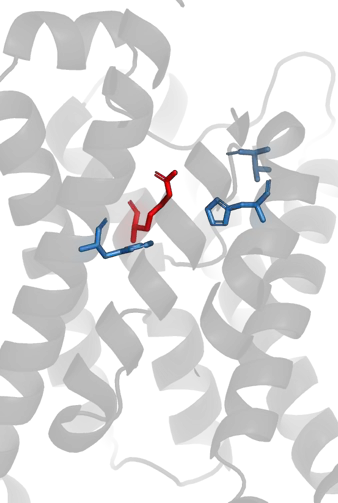


**R-231**

**T-223**

**H-216**

**F-51**


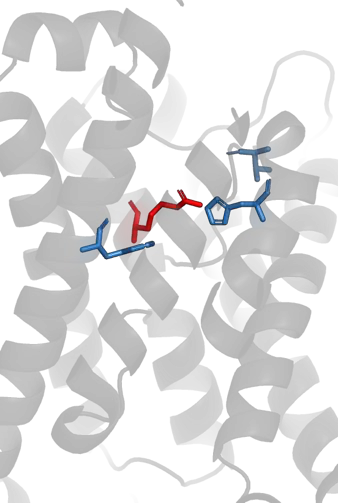


**T-223**

**R-228**

**H-213**

**F-49**


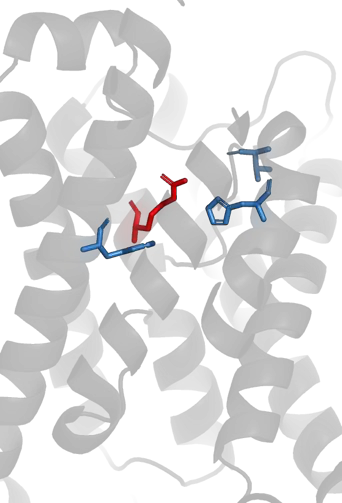


**T-223**

**R-228**

**H-213**

**F-49**

**Figure S3.** Conformation of sidechains of NPA boxes and ar/R motif in AtPIP2;1,

AtPIP2;3 and AtPIP2;3H147Y-F157S.

**SiPIP2;1** SLVRALLYIVAQCLGAICGVGLVKAFQ-SAYFDRYGGGANSLASGYSRG **HvPIP2;5** SLVRALLYMVAQCLGAMCGVGLVKAFQ-SAYFVRYGGGANTLAAGYSKG **HvPIP2;3** SLVRAVLYIIAQCLGAICGVGLVKGFQ-SAFYVRYGGGANELSAGYSKG **HvPIP2;4** SLVRAVLYIIAQCLGAICGVGLVKGFQ-SAFYVRYGGGANELSAGYSKG **HvPIP2;1** SLIRALLYIIAQCLGAICGVGLVKGFQ-SSYYVRYGGGANELSAGYSKG **SiPIP2;4** SLVRAVLYIIAQCLGAICGVGLVKGFQ-SAYFVRYGGGANELSAGYSKG **AtPIP2;5** TLVRAVMYMVAQCLGAICGVALVKAFQ-SAYFTRYGGGANGLSDGYSIG **AtPIP2;6** SLVRAVSYMVAQCLGATCGVGLVKVFQ-STYYNRYGGGANMLSDGYNVG **AtPIP2;2** SLIRAVLYMVAQCLGAICGVGFVKAFQ-SSYYDRYGGGANSLADGYNTG **AtPIP2;3** SLR-AVLYMVAQCLGAICGVGFVKAFQ-SSHYVNYGGGANFLADGYNTG **AtPIP2;1** SLPRALLYIIAQCLGAICGVGFVKAFQ-SSYYTRYGGGANSLADGYSTG **AtPIP2;4** SLVRTVLYIVAQCLGAICGCGFVKAFQ-SSYYTRYGGGANELADGYNKG **NtPIP2;1** SLVRAIMYMLAQCLGAICGCGLVKAFQ-KAYYVKYGGGANTLNDGYNTG **AtPIP2;7** SLVRALGYMIAQCLGAICGVGFVKAFM-KTPYNTLGGGANTVADGYSKG **AtPIP2;8** SLPRAVAYMVAQCLGAICGVGLVKAFM-MTPYKRLGGGANTVADGYSTG **SiPIP2;5** SLVRAALYIVAQCLGAICGAGLVRAFHGTSSYLRHGGGANELAAGYSKG **HvPIP2;2** SLLRAVMYIVAQCAGGIVGAGIVKGIM-KDAYQANGGGANMVASGFSRG **SiPIP2;7** SLLRTVLYIVAQCLGAICGVAIVKGIT-GDQYSLLGGGANSVADGFSVV

**Figure S4.** Alignment of deduced partial amino acid sequences of PIP2 aquaporins in Arabidopsis, tobacco, barley and foxtail millet. The red boxes indicate the position of critical amino acids found in this study. Blue color: CO_2_-permeable aquaporins, red color: no or low CO_2_-permeable aquaporins.

HvPIP2;3 HvPIP2;4


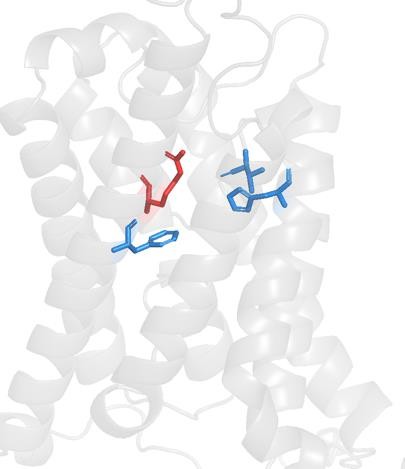

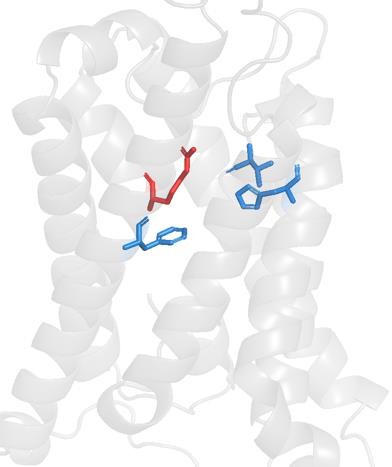


**R-235**

**T-229**

**R-235**

**T-229**

**H-220**

**H-220**

**F-52**

**F-52**

**Figure S5.** Three dimensional positions of sidechains of

ar/R motif in HvPIP2;3 and HvPIP2;4.

HvPIP2;2 SiPIP2;7 AtPIP2;7 AtPIP2;8

NtPIP2;1

**AtPIP2;4**

AtPIP2;1

AtPIP2;3 AtPIP2;2

AtPIP2;5 AtPIP2;6 HvPIP2;5 SiPIP2;1 SiPIP2;5 SiPIP2;4 HvPIP2;1 HvPIP2;3 HvPIP2;4

**Figure S6.** Phylogenetic tree of PIP2 aquaporins of Arabidopsis, tobacco (*Nicotiana tabacum*), barley (*Hordeum vulgare*) and foxtail millet (*Setalia italica*). Blue: CO_2_/H_2_O dual-permeable aquaporins, Red: no or low CO_2_-permeable/H_2_O- permeable aquaporins, **Green** (bolded for enhancing readability for individuals with color vision impairment): CO_2_-permeable/no or low H_2_O-permeable aquaporins. Phylogenetic tree was generated by ClustalW with Neighbour Joining method using the full-length amino acid sequences. The Gene Identifier codes are AtPIP2;1, At3g53420; AtPIP2;2, At2g37170; AtPIP2;3, At2g37180; AtPIP2;4, At5g60660; AtPIP2;5, At3g54820; AtPIP2;6, At2g39010; AtPIP2;7, At4g35100; AtPIP2;8, At2g16850; NtPIP2;1, AF440272; HvPIP2;1, AB219366; HvPIP2;2, AB377269; HvPIP2;3, AB275280; HvPIP2;4, AB219525; HvPIP2;5, AB377270; SiPIP2;1, 004956113.1; SiPIP2;4, 004953172.1; SiPIP2;5, 012698174.2; SiPIP2;7,

004957505.1.

TM3

Loop C

TM4


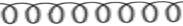

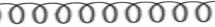


Arabidopsis PIP2;1 (P43286) YIIAQCLGAICGVGFVKAFQSS*YYTRYGGG ANS*LADGYSTGTGLAAEIIGTFVLVYTVFSA

Synechococcus AqpZ_(K9RY94) YIVAQVIGAVIAGGLVYLIASGRGGGFVLGGSNPLATNG FGAHSPGGYGLVAALITEVVMTFMFLLVILGA

Human AQP8 (O94778) YIIAQCVGAIVATAILSGISPEERFWNASG AAFVTVQEQGQVAGALVAEIILTTLLALAVCMG

Rat AQP8 (P56405) YIIAQCVGAIVATAILSGISPEERFWNASG AAFAIVQEQEQVAEALGVEIVMTMLLVLAVCMG

Human AQP1 (P29972) YIIAQCVGAIVATAILSGITSSLTGNSLG RNDLADGVNSGQGLGIEIIGTLQLVLCVLAT

Rat AQP1 (P29975) YIIAQCVGAIVATAILSGITSSLLENSLG RNDLARGVNSGQGLGIEIIGTLQLVLCVLAT

Bovine AQP1 (P47865) YIIAQCVGAIVATAILSGITSSLPDNSLG LNALAPGVNSGQGLGIEIIGTLQLVLCVLAT

Zebrafish AQP1a1 (Q6NZ72) YILAQMIGATVASAIVLGVSK---GDALG LNQIHTDISAGQGVGIELLATFQLVLCVLAT

Human AQP2 (P41181) YVAAQLLGAVAGAALLHEITPADIRGDLA VNALSNSTTAGQAVTVELFLTLQLVLCIFAS

Bovine AQP2 (P79099) YVAAQLLGAVAGAALLHEITPPAIRGDLA VNALNNNSTAGQAVTVELFLTLQLVLCIFAS

Rat AQP2 (P34080) YVAAQLLGAVAGAAILHEITPVEIRGDLA VNALHNNATAGQAVTVELFLTMQLVLCIFAS

Human AQP5 (P55064) YVAAQLVGAIAGAGILYGVAPLNARGNLA VNALNNNTTQGQAMVVELILTFQLALCIFAS

Rat AQP5 (P47864) YVAAQLVGAIAGAGILYWLAPLNARGNLA VNALNNNTTPGKAMVVELILTFQLALCIFAS

Human AQP6 (Q13520) YVAAQLVGATVGAALLYGVMPGDIRETLG INVVRNSVSTGQAVAVELLLTLQLVLCVFAS

Rat AQP6 {Q9WTY0) YIAAQLAGATVGAALLYGVTPGGVRETLG VNVVHNSTSTGQAVAVELVLTLQLVLCVFAS

Human AQP4 (P55087) YIAAQCLGAIIGAGILYLVTPPSVVGGLG VTMVHGNLTAGHGLLVELIITFQLVFTIFAS

Bovine AQP4 (O77750) YIAAQCLGAIIGAGILYLVTPPSVVGGLG VTTVHGNLSAGHGLLVELIITFQLVFTIFAS

Rat AQP4 (P47863) YITAQCLGAIIGAGILYLVTPPSVVGGLG VTTVHGNLTAGHGLLVELIITFQLVFTIFAS

Human AQP3 (Q92482) YTLAQTLGAFLGAGIVFGLYYDAIWHFADNQLFVSGPNGTAGIFATYPSGHLDMINGFFDQFIGTASLIVCVLAI Bovine AQP3 (Q08DE6) YTLAQTLGAFLGAGIIFGLYYDAIWAFANNQLIVSGPNGTAGIFATYPSGHLDMVNGFFDQFIGTASLIVCVLAI Rat AQP3 (P47862) YTLAQTLGAFLGAGIVFGLYYDAIWAFAGNELVVSGPNGTAGIFATYPSGHLDMVNGFFDQFIGTAALIVCVLAI Human AQP10 (Q96PS8) YILVQLLSAFCASGATYVLYHDALQNYTGGNLTVTGPKETASIFATYPAPYLSLNNGFLDQVLGTGMLIVGLLAI Human AQP9 (O43315) YVGAQFLGAFVGAATVFGIYYDGLMSFAGGKLLIVGENATAHIFATYPAPYLSLANAFADQVVATMILLIIVFAI Rat AQP9 (P56627) YVGAQFLGAFVGAATVFGIYYDGLMAFAGGKLLVVGENATAFIFATYPAPFISTPGAFVDQVVSTMFLLLIVFAM Human AQP7 (O14520) YVLGQFLGSFLAAATIYSLFYTAILHFSGGQLMVTGPVATAGIFATYLPDHMTLWRGFLNEAWLTGMLQLCLFAI Rat AQP7 (P56403) YVLGQFLGSFLAAATTYLIFYGAINHYAGGELLVTGPKSTANIFATYLPEHMTLWRGFVDEVFVTGMLQLCIFAI Human AQP11 (Q8NBQ7) RLLAQLVSALCSRYCTSALWSLGLTQYHVSE RSFACKNPIRVDLLKAVITEAVCSFLFHS

Rat AQP11 (Q8CHM1) RLMAQLVSALCSRYCISALWSLSLTKYHFDE RILACRNPINTDISKAIIIEAICSFIFHS

Human AQP12 (Q8IXF9) KLAAQGLGMQAACTLMRLCWAWELSDLHLLQS LMAQSCSSALRTSVPHGALVEAACAFCFHL

Rat AQP12a (D4A9T6) KLLAQVLGAQAACALTQRCWTWELSELHLLQS LMAVHCSSTLRTSVLQGTLVEGACTFLFHL

**Figure S7.** Amino acid sequence alignment of transmembrane domain (TM) 3, loop C and TM4 of aquaporins of human, rat, bovine, Synechococcus sp. PCC7942 AqpZ and *Arabidpsis thaliana* PIP2;1. Asterisks indicate Tyr-150 and Ser-160 in Arabidopsis PIP2;1. Accession numbers are shown in parentheses.

0.02

*PIP2;4/ACTIN*

a

0.2


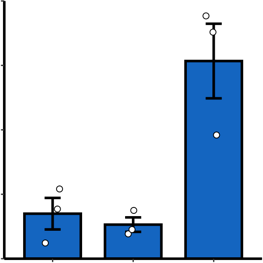


b

b


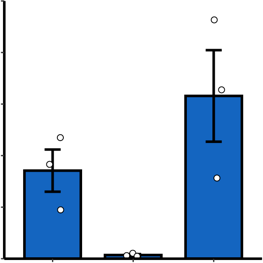


ab

b

*PIP2;2/ACTIN*

a

0.04


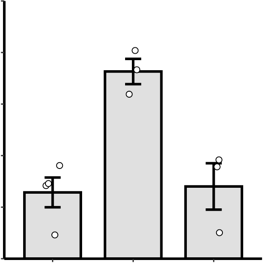


a

b

b

*PIP2;3/ACTIN*

0.010

0.01

*PIP2;1/ACTIN*

0.1

0.02

0.005

0.00

0.03

0.0

WL MCP GC WL MCP GC

0.8


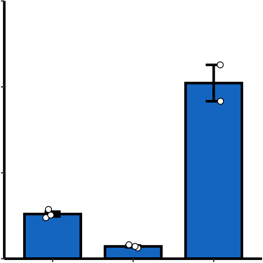


a

b

b


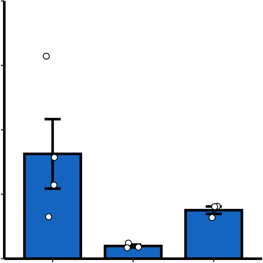


a

ab

b

0.00

0.3

WL MCP GC

0.000

0.004


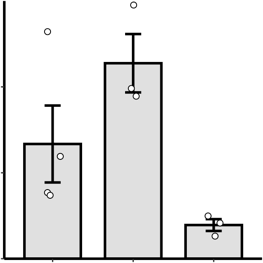


ab

a

b


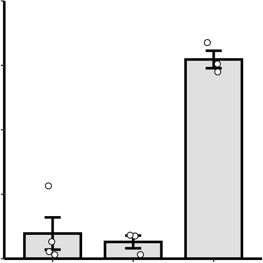


a

b

b

WL MCP GC


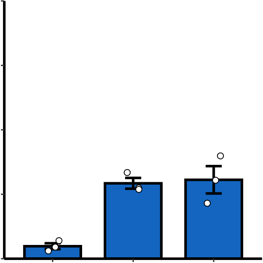


a

a

b

0.02

*PIP2;5/ACTIN*

0.01

0.4

0.2

0.1

*PIP2;7/ACTIN*

0.002

0.00

*PIP2;6/ACTIN*

*PIP2;8/ACTIN*

0.0

WL MCP GC WL MCP GC

0.0

WL MCP GC

0.000

WL MCP GC

**Figure S8.** Relative expression of *AtPIP2* genes relative to that of *ACTIN* in whole leaf (WL), mesophyll cell protoplasts (MCP) and guard cell-enriched epidermis (GC) of *Arabidopsis thaliana* ecotype Columbia*.* Blue bars and white bars indicate CO_2_-permeating and CO_2_-non-permeating aquaporins, respectively. Different letters show the significant difference (α = 0.05) by the Tukey- Kramer multiple comparison test. The relative expression level was calculated using the ΔΔ*C*_t_ method with the reference gene (*ACTIN2/8*) as an internal standard.
